# Increasing Antiproliferative Properties of Endocannabinoids in N1E-115 Neuroblastoma Cells through Inhibition of Their Metabolism

**DOI:** 10.1371/journal.pone.0026823

**Published:** 2011-10-27

**Authors:** Laurie Hamtiaux, Laurie Hansoulle, Nicolas Dauguet, Giulio G. Muccioli, Bernard Gallez, Didier M. Lambert

**Affiliations:** 1 Medicinal Chemistry, Cannabinoid and Endocannabinoid Research Group, Louvain Drug Research Institute, Université Catholique de Louvain, Brussels, Belgium; 2 de Duve Institute, Université Catholique de Louvain, Brussels, Belgium; 3 Bioanalysis and Pharmacology of Bioactive Lipids Laboratory, Louvain Drug Research Institute, Université Catholique de Louvain, Brussels, Belgium; 4 Biomedical Magnetic Resonance, Louvain Drug Research Institute, Université Catholique de Louvain, Brussels, Belgium; University of Medicine and Dentistry of New Jersey, United States of America

## Abstract

The antitumoral properties of endocannabinoids received a particular attention these last few years. Indeed, these endogenous molecules have been reported to exert cytostatic, apoptotic and antiangiogenic effects in different tumor cell lines and tumor xenografts. Therefore, we investigated the cytotoxicity of three *N*-acylethanolamines – *N*-arachidonoylethanolamine (anandamide, AEA), *N*-palmitoylethanolamine (PEA) and *N*-oleoylethanolamine (OEA) - which were all able to time- and dose-dependently reduce the viability of murine N1E-115 neuroblastoma cells. Moreover, several inhibitors of FAAH and NAAA, whose presence was confirmed by RT-PCR in the cell line, induced cell cytotoxicity and favored the decrease in cell viability caused by *N*-acylethanolamines. The most cytotoxic treatment was achieved by the co-incubation of AEA with the selective FAAH inhibitor URB597, which drastically reduced cell viability partly by inhibiting AEA hydrolysis and consequently increasing AEA levels. This combination of molecules synergistically decreased cell proliferation without inducing cell apoptosis or necrosis. We found that these effects are independent of cannabinoid, TRPV1, PPARα, PPARγ or GPR55 receptors activation but seem to occur through a lipid raft-dependent mechanism. These findings further highlight the interest of targeting the endocannabinoid system to treat cancer. More particularly, this emphasizes the great potential benefit of designing novel anti-cancerous therapies based on the association of endocannabinoids and inhibitors of their hydrolysis.

## Introduction

Since the identification of Δ^9^-tetrahydrocannabinol as the main psychoactive constituent of *Cannabis sativa* [1], a notable amount of studies have been carried out in the cannabinoid field in order to elucidate the physiological functions of the endocannabinoid system and its potential relevance in health and diseases. To date, two types of G protein-coupled receptors playing a role in the endocannabinoid system have been cloned, the CB_1_ and CB_2_ cannabinoid receptors [2], [3]. The two major endocannabinoids, *N*-arachidonoylethanolamine (AEA, anandamide) and 2-arachidonoylglycerol (2-AG), are endogenous bioactive lipids activating the cannabinoid receptors [4]. However, it is now well established that other receptors, like the vanilloid receptor 1 (TRPV1) [5], two G protein-coupled receptors - GPR55 and GPR119 [6] - and the peroxisome proliferator-activated receptors (PPAR's) [7], are involved in the pharmacological effects of cannabinoids.

Along with the endocannabinoids, other endogenous mediators belonging to the *N*-acylethanolamine family and exerting cannabimimetic actions are known. These “endocannabinoid-like” compounds, including *N*-palmitoylethanolamine (PEA) and *N*-oleoylethanolamine (OEA), do not bind the CB_1_ and CB_2_ cannabinoid receptors although they share with AEA common metabolic pathways as well as molecular targets such as PPAR's [8].

Several enzymes tightly regulate endocannabinoid levels. The best characterized is the fatty acid amide hydrolase (FAAH) [9] which is responsible for the hydrolysis of the *N*-acylethanolamines AEA, PEA and OEA. The *N*-acylethanolamine-hydrolyzing acid amidase (NAAA) is another amidase catalyzing the same reaction, although at a more acidic pH [10]. The major enzyme responsible for the degradation of 2-AG is the monoacylglycerol lipase (MAGL) [11].

During these last few years endocannabinoid antitumoral effects received a particular attention, including for their ability to decrease proliferation and viability of different cancer cell lines both *in vitro* and *in vivo*. The mechanisms at the origin of these effects are multifarious and implicate growth arrest, induction of apoptosis, angiogenesis inhibition and antimetastatic effects [12]. AEA was reported to inhibit human breast cancer cell proliferation through the CB_1_ receptor without inducing apoptosis. 2-AG, but not PEA, also showed analogous antiproliferative effects [13]. Similar results were obtained on human prostatic cancer cell lines on which, acting through the CB_1_ receptor, AEA induced massive cell apoptosis and necrosis [14]. But cannabinoids can also exert antitumoral effects by acting via the CB_2_ receptor [15], the TRPV1 receptor [16] or through a combined activation of both cannabinoid and vanilloid receptors [17]. In addition, though PEA has no antiproliferative properties by itself, it was shown to enhance AEA activity at vanilloid TRPV1 receptor [18] and to enhance AEA-induced cytostatic effects mediated by the CB_1_ cannabinoid receptor [19]. An enzymatic approach has also been described in which arachidonoyl-serotonin (AA-5-HT), a blocker of endocannabinoid enzymatic hydrolysis, was proved to be effective in reducing cell proliferation and tumor development [20]. Together these studies show the implication of the endocannabinoid system in malignancy and suggest the therapeutic benefits that would offer its modulation in the treatment of cancer.

Neuroblastoma is the most frequent extracranial solid tumor of childhood and continues to carry a poor prognosis [21]. Lack of efficacy of treatments made it necessary to find new therapeutic strategies. Several cells lines, including N1E-115 (as shown by Mundy et al. [22], Grimbly et al. [23] and Favier et al. [24]), have been used as neuroblastoma model for studying proliferation and cell toxicity. Furthermore, because we wanted to study the role of the endocannabinoid system in neuroblastoma cell viability we opted for the N1E-115 cell line that was already shown to express CB_1_ cannabinoid receptors [25]. Thus, we further characterized here the endocannabinoid system in the N1E-115 murine neuroblastoma cell line and investigated the cytotoxicity of endocannabinoids, their metabolism inhibition and the potential implication of cannabinoid and non-cannabinoid receptors in cell viability. By studying the mechanisms of activity, we point up the possibility of enhancing the antiproliferative properties of the endocannabinoid anandamide by inhibiting its degradation using the selective FAAH inhibitor URB597.

## Materials and Methods

### 1. Drugs

Anandamide, *N*-palmitoylethanolamine and *N*-oleoylethanolamine as well as arachidonic acid, palmitic acid and oleic acid were all from Tocris Bioscience. The enzyme inhibitors URB597, CAY10402 and CAY10499 were bought from Cayman Europe and MAFP from Tocris Bioscience. CCP (*N*-cyclohexanecarbonylpentadecylamine) was kindly synthesized by Coco N. Kapanda (Université catholique de Louvain, Belgium). All the receptor antagonists (AM251, capsazepine, GW6471, T0070907 and (-)-cannabidiol) were purchased from Tocris Bioscience and the lipid raft disruptor methyl-β-cyclodextrin was from Sigma-Aldrich. The endocannabinoids, fatty acids, enzyme inhibitors and receptor antagonists were prepared in DMSO at a stock concentration of 2×10^−2^ M and diluted in media for the experiments conducted on cells. Dilutions of methyl-β-cyclodextrin were performed in PBS using a stock concentration of 2×10^−1^ M. The final concentration of DMSO was kept below 0.2%. [^3^H]-anandamide (60 Ci/mmol) and [^3^H]-PEA (20 Ci/mmol) were purchased from American Radiolabeled Chemicals (St Louis, MO, USA).

### 2. Cell culture

The murine neuroblastoma cell line N1E-115 was obtained from the American Type Culture Collection and was routinely cultured in Dulbecco's modified Eagle medium D-MEM/NUT mix F12 (1/1) medium supplemented with 10% fetal bovine serum, 100 UI/ml penicillin, 100 mg/ml streptomycin and 2mM L-glutamine. Cells were maintained at 37°C in a humidified atmosphere of 5% CO_2_.

### 3. MTT cell viability assay

The effect on cell viability of the different treatments was measured using MTT assay, which is based on the transformation of 3-(4,5-dimethylthiazol-2-yl)-2,5diphenyltetrazolium bromide (MTT) in formazan crystals by the mitochondrial succinate dehydrogenase of viable cells. Cells were plated in 96-well plates at a density of 2000 cells/well in D-MEM medium supplemented with 10% serum. After 5h of incubation at 37°C in a 5% CO_2_ humidified atmosphere, test compounds diluted in culture medium were added in each well for 24h, 48h or 72h. The medium was then removed and 100 µl of MTT solution (0.3 mg/ml) were added for a 2h incubation. The MTT solution was removed, replaced by 100 µl DMSO to dissolve the crystalline formazan product and the absorbance was read at 570nm (with a reading at 650nm as reference) using a microplate spectrophotometer. For the treatments with the receptor antagonists and methyl-beta-cyclodextrin, only the 72h time point was considered and the antagonists were incubated 1h before the beginning of the cytotoxic treatment.

### 4. Cell proliferation assay

The antiproliferative properties of tested molecules were measured by [^3^H]-thymidine incorporation assay and performed in microwells. Cells were incubated with the drugs for 24h and [methyl-^3^H]thymidine (0.5 mCi/well) was added during the last 8h of a 24h treatment. During each cell division [^3^H]-thymidine is incorporated into the cell DNA and at the end of the incubation the amount of radioactivity incorporated by the cells was measured after filtration by liquid scintillation.

### 5. Cell death

#### 5.1. Caspase 3 activity

Cell death by apoptosis was assessed by measurement of caspase 3 activity monitored by cleavage of a specific peptide substrate Asp-Glu-Val-Asp-AFC (DEVD-AFC) according to the FluorAce apopain assay kit (Bio-Rad). After 24h treatment with the cytotoxic agent, the cells were collected, centrifuged and washed with PBS before lysis. After a second centrifugation, the supernatant was incubated with the peptide substrate and the cleavage was measured after 0, 30, 60, 90 and 120 min using a fluorescence spectrometer (375nm excitation, 530nm emission). Cells treated with 10 µM of sanguinarine for 4h were used as positive control.

#### 5.2. Annexin-V/Propidium iodide staining

Detection and quantification of apoptosis was performed by the analysis of phosphatidylserine on the outer leaflet of apoptotic cell membranes using Annexin-V-Fluorescein. Propidium iodide was used for the differentiation from necrotic cells. Cells were incubated for 24h with the cytotoxic treatment before being stained with the Roche Annexin-V-FLUOS Staining kit (Mannheim, Germany) following the manufacturer's instructions. Cells treated with 5 µM of camptothecin were used as positive control. Cells were examined using a fluorescence microscope from Optika (Ponteranica, Italy). Pictures were taken with a Moticam 2300 from Motic (Hong Kong, China).

### 6. Cell cycle analysis

Cell cycle analysis was performed on N1E-115 cells plated in 6-well plates, initially seeded at a density of 50, 100 or 150×10^3^ cells/well and incubated for 24h, 48h or 72h respectively with vehicle or both AEA and URB597 at 20 µM. When specified, cells were synchronized with 30ng/ml nocodazole for 14h prior to the addition of the tested compounds. After treatment, cells were harvested by trypsinization, washed with PBS, pelleted and fixed by rapid submersion in ice-cold 80% ethanol with vigorous vortexing. After overnight fixation at −20°C, cells were washed with PBS, pelleted, resuspended and incubated for 20min in a saponin-based permeabilization solution containing 1% BSA, 0.2 mg/ml Ribonuclease A and 20 µg/ml propidium iodide. Data were collected on a LSRFortessa (BD Biosciences) and analyzed with the FlowJo software (Treestar).

### 7. Cell morphology

Cells were observed after various incubation times under an inverted microscope from Optika (Ponteranica, Italy). Pictures were taken with a Moticam 2300 from Motic (Hong Kong, China). Light microscopic evaluation was performed using a magnification of 400x.

### 8. Reverse transcriptase-polymerase chain reaction

Total RNA was extracted from the cultured cells with the TriPure Isolation reagent (Roche). To measure mRNA expression, reverse transcription was performed using the Reverse Transcription System (Promega) and the generated cDNA was amplified by PCR using the primers mentioned in the Table 1. Polymerase chain reactions were performed according to the following parameters: 95°C for 10min, 95°C for 3s, 60°C for 26s, and 72°C for 10s (45 cycles). After amplification, agarose gel electrophoresis was used to detect the expression of the genes.

**Table 1:** Primer sequences used for PCR amplification.

RPL19	F: gaaggtcaaagggaatgtgttca		
	R: ccttgtctgccttcagcttgt		
FAAH	F: gagatgtatcgccagtccgt	GPR55	F: atttggagcagaggcacgaacatga
	R: acaggcaggcctataccctt		R: agtggcgatatagtccagcttcct
NAAA	F: ggttttatccctgtttcctgtttat	TRPV1	F: aactcttacaacagcctgtattccaca
	R: tttttgacaatacatcaccttcagct		R: aagacagccttgaagtcatagttct
CB_1_	F: ctgatgttctggatcggagtc	PPARα	F: caacggcgtcgaagacaaa
	R: tctgaggtgtgaatgatgatgc		R: tgacggtctccacggacat
CB_2_	F: tgacaaatgacacccagtcttct	PPARγ	F: ctgctcaagtatggtgtccatga
	R: actgctcaggatcatgtactcctt		R: tgagatgaggactccatctttattca

### 9. Enzymatic activity and inhibition

#### 9.1. On cell homogenates

In order to detect the presence of *N*-acylethanolamine enzymatic hydrolysis, glass tubes containing increasing amounts of cell homogenates (165 µl, 10mM Tris-HCl, 1mM EDTA, pH 7.4) and 10 µl of DMSO were incubated for 10min at 37°C with the radiolabeled substrate (either [^3^H]-anandamide or [^3^H]-*N*-palmitoylethanolamine (25 µl, 50000 dpm)). Reactions were stopped by rapidly placing the tubes in ice-cold water followed by the addition of cold chloroform-methanol (1∶1 v/v, 400 µl). After centrifugation (850*g*, 5min, 4°C) the radioactivity in the aqueous phase (200 µl) was counted by liquid scintillation (UltimaGold from Perkin-Elmer). To estimate the inhibition potential on N1E-115 cell homogenates of the inhibitors, a set amount of homogenate was chosen (25 µg of protein/tube) and compounds in DMSO (10 µl), or DMSO alone for control, were added. As control for chemical hydrolysis, dpm values obtained for tubes containing buffer instead of proteins were systematically subtracted.

#### 9.2. On living cells

Cells were seeded 24h before treatment at a concentration of 10^5^ cells/well in a 24-well plate. The medium was removed and replaced by 200 µl of fresh medium 30min before the beginning of the experiment. Test compounds were added to each well (150 µl) followed by the radiolabeled substrate (50 µl, 50000 dpm) and the plate was incubated 10min at 37°C in a 5% CO_2_ humidified atmosphere. Then, the reaction was stopped by adding 400 µl of cold methanol on ice. After scraping the wells, a volume of 600 µl was removed and placed in a glass tube where 300 µl chloroform were added. The tubes were centrifuged (850*g*, 10min, 4°C) and a 400 µl aliquot of the aqueous upper phase was used to measure the radioactivity by liquid scintillation (UltimaGold from Perkin-Elmer). Cells incubated with vehicle (DMSO) were used as control and wells containing no cells were used as blank.

### 10. *N*-acylethanolamine quantification by HPLC-MS

Cells (10^7^ cells/flask) were seeded in 10% FBS media for 12h prior to the incubation (2h) with the drugs, or vehicle, in 1% FBS media. The cells were then recovered and the lipids extracted using a CHCl_3_ – MeOH – H_2_0 (10∶5∶2.5) mixture. Following centrifugation, the organic layer was recovered, dried under a stream of N_2_ and purified by solid-phase extraction using silica, followed by elution with an EtOAc-Acetone (1∶1) solution [26], [27]. The resulting lipid fraction was analysed by HPLC-MS using a LTQ Orbitrap mass spectrometer (ThermoFisher Scientific) coupled to an Accela HPLC system (ThermoFisher Scientific) [28]. Analyte separation was achieved using a C-18 Supelguard pre-column and a Supelcosil LC-18 column (3 µM, 4×150 mm) (Sigma-Aldrich). Mobile phases A and B were composed of MeOH-H_2_O-acetic acid 75∶25∶0.1 (v/v/v) and MeOH-acetic acid 100∶0.1 (v/v), respectively. The gradient (0.5 ml/min) was designed as follows: transition from 100% A to 100% B linearly over 15min, followed by 10min at 100% B and subsequent re-equilibration at 100% A. We performed MS analysis in the positive mode with an APCI ionisation source. The capillary and APCI vaporiser temperatures were set at 250°C and 400°C, respectively. *N*-acylethanolamines were quantified by isotope dilution using their respective deuterated standards with identical retention. The calibration curves were generated as previously described [26], and the data were normalised by cell number.

### 11. Statistical analysis

Values were expressed as mean ± SEM. Statistical analysis was performed by ANOVA or by unpaired Student's *t* test.

## Results

### 1. *N*-acylethanolamines time- and dose-dependently decrease N1E-115 cell viability

**Figure 1:**
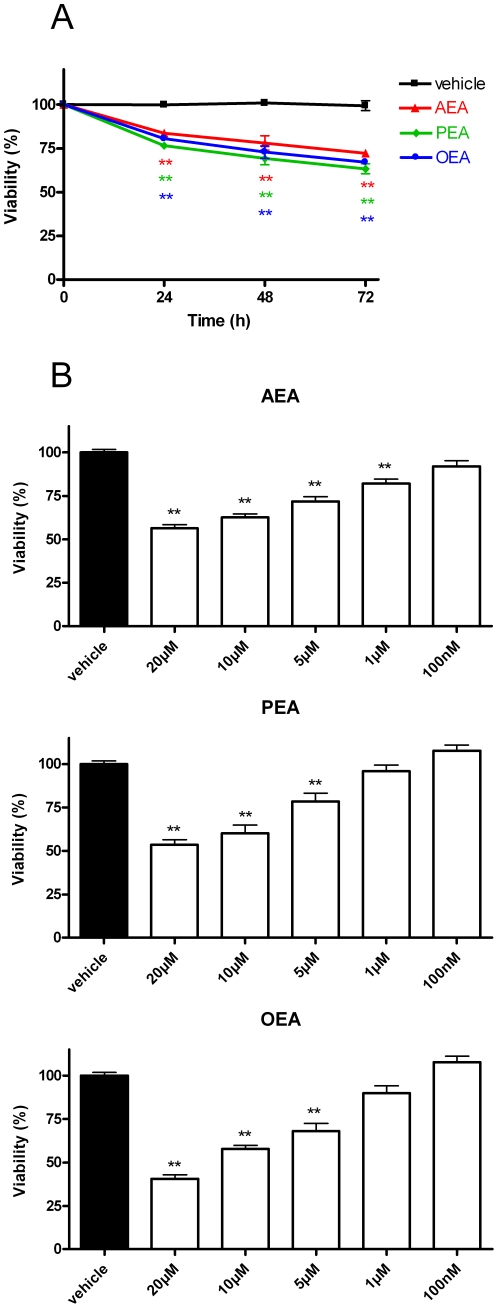
*N*-acylethanolamines induce N1E-115 neuroblastoma cell cytotoxicity. *N*-acylethanolamines AEA, PEA and OEA time- (*A*) and dose-dependently (*B*) decrease N1E-115 cell viability. Cells were seeded 5h before treatment (2000 cells/well in microwells) and incubated with increasing concentrations of *N*-acylethanolamines. After 24h, 48h or 72h of treatment, cytotoxicity was assessed by a MTT test. Data are expressed as percentage of the vehicle control and are the mean of three experiments performed in quintuplicate. Significantly different (**P<0.01) from vehicle incubation.

We assessed the effect of the endocannabinoid AEA and related bioactive lipids – PEA and OEA – on the viability of N1E-115 cells, a neuroblastoma cell line, using a MTT assay. We observed that already after 24h of treatment, AEA, PEA and OEA (at 10 µM) are equally effective in reducing the number of metabolically active cells, expressed as cell viability, in comparison to vehicle, and that this effect is amplified after 48h and 72h of incubation (Fig. 1A). We then tested increasing concentrations of *N*-acylethanolamines (from 100nM up to 20 µM) to study the dose-dependent character of the observed effect. Here, we show that, after 72h of treatment (*i.e*. the time at which the effects were most apparent), AEA, PEA and OEA decrease neuroblastoma cell viability in a dose-dependent manner (Fig. 1B). To ensure that the cytotoxicity was not due to the *N*-acylethanolamine fatty acid metabolites – *i.e.* arachidonic acid, palmitic acid and oleic acid for AEA, PEA and OEA respectively – we tested these fatty acids at 0.1 µM, 1 µM and 10 µM. Although a little effect was observed for palmitic acid and oleic acid (see Fig. S1) this was not sufficient to account for the *N*-acylethanolamine-mediated reduction of cell viability.

### 2. *N*-acylethanolamine enzymatic degradation

**Figure 2:**
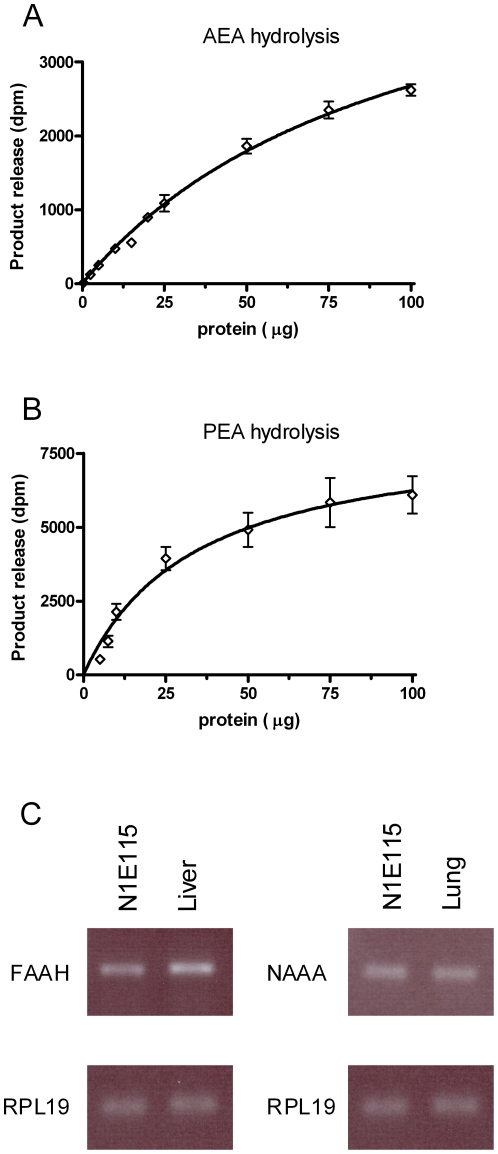
N1E-115 cells efficiently hydrolyze *N*-acylethanolamines. Enzymatic activities for AEA (*A*) and PEA (*B*) hydrolysis were measured in N1E-115 cell homogenates using [^3^H]-AEA and [^3^H]-PEA, respectively. Data are the mean of three experiments performed in duplicate. N1E-115 cells express *N*-acylethanolamines degrading enzymes FAAH and NAAA (*C*). Detection of mRNA was performed by RT-PCR using respectively mouse liver and lung as control and RPL19 as house keeping gene (blot representative of three).

Since the aim of this work was to study the effect of *N*-acylethanolamines on N1E-115 cell viability, we found primordial to determine the rate of hydrolysis of these bioactive lipids by the cells. Thus, using [^3^H]-AEA and [^3^H]-PEA, we found that N1E-115 cell homogenates significantly hydrolyze *N*-acylethanolamines (Fig. 2A and 2B). Accordingly, we detected in N1E-115 cells the mRNA coding for the two major *N*-acylethanolamine degrading enzymes, the fatty acid amide hydrolase (FAAH) and the *N*-acylethanolamine-hydrolyzing acid amidase (NAAA) (Fig. 2C). Consistent with the results obtained with homogenates (at pH 7.4), we were also able to detect the hydrolysis of [^3^H]-AEA and [^3^H]-PEA when using N1E-115 cells in culture (Table 2). Note that the hydrolysis of OEA could not be directly tested as no radiolabeled analogue is commercially available.

**Table 2:** Inhibition of *N*-acylethanolamine hydrolysis by N1E-115.

		Hydrolysis inhibition (%± SEM)			
		AEA hydrolysis		PEA hydrolysis	
		Cell homogenates	Intact cells	Cell homogenates	Intact cells
URB597	10 µM	***100***±***0.2***	***85***±***2.9***	***96***±***1.9***	***73***±***6.5***
	1 µM	***99***±***0.3***	***86***±***2.0***	***87***±***3.4***	***74***±***4.3***
CAY10402	10 µM	***100***±***0.5***	***62***±***6.2***	***89***±***2.5***	***66***±***7.0***
	1 µM	***100***±***0.7***	***43***±***7.5***	***85***±***1.4***	***58***±***7.6***
MAFP	10 µM	***100***±***0.3***	***86***±***2.9***	***89***±***1.8***	***63***±***6.0***
	1 µM	***100***±***0.2***	***92***±***3.1***	***84***±***1.9***	***62***±***5.3***
CAY10499	10 µM	***100***±***0.5***	***93***±***2.5***	***88***±***1.7***	***68***±***3.5***
	1 µM	***90***±***0.6***	***81***±***1.8***	***80***±***2.2***	***55***±***5.1***
CCP	10 µM	***3***±***2.5***	***9***±***4.0***	***7***±***3.1***	***22***±***4.9***
	1 µM	***6***±***2.0***	***3***±***3.4***	***5***±***3.7***	***9***±***5.6***

FAAH inhibitors (URB597, CAY10402), NAAA inhibitors (CCP) and dual inhibitors of FAAH and MAGL (MAFP, CAY10499) were tested at concentrations of 1 and 10 µM on cell homogenates (25 µg protein, pH 7.4) and on intact cells (10^5^ cells/well, seeded 24h before) in culture medium. Data are the mean of three experiments and are expressed as percentage of the control containing vehicle instead of the inhibitors.

As enzymatic activities for the hydrolysis of *N*-acylethanolamines were detected, we sought to determine whether it would be possible to block this hydrolysis in order to increase the effects on cell viability observed with AEA, PEA and OEA.

### 3. Inhibition of *N*-acylethanolamine degradation

We tested at 1 µM and 10 µM several drugs able to decrease *N*-acylethanolamine hydrolysis either by inhibiting selectively FAAH (URB597 and CAY10402) or NAAA (CCP), or by concomitant inhibition of FAAH and MAGL (MAFP and CAY10499) (see Fig. S2). The inhibition assays were performed either on total cell homogenates or on cells in culture (Table 2) to confirm that the inhibitors reach their targets in culture conditions.

As expected, URB597, CAY10402, MAFP and CAY10499 all inhibit AEA hydrolysis in homogenates and cultured cells. Note that the inhibition is slightly less pronounced in the later case, especially for CAY10402 which at 1 µM inhibited 43±7.5% of AEA hydrolysis in intact cells compared to 100±0.7% on cell homogenates. The NAAA inhibitor, CCP, had almost no effect on AEA hydrolysis both in homogenates and in intact cells.

The proposed metabolic pathways for PEA and AEA are relatively similar. Accordingly, the inhibitors similarly affected PEA and AEA hydrolysis, although PEA hydrolysis was less sensitive to inhibition. Surprisingly we did not observe an inhibition of PEA hydrolysis when using CCP in homogenates or only a slight decrease in intact cells (22±4.9% inhibition at 10 µM). This could be explained by the fact that FAAH can also hydrolyze PEA and thus that FAAH could compensate for the decrease in NAAA activity upon inhibition by CCP [29]. Another possible explanation is that the assay was performed on homogenates at physiological pH while it is known that NAAA activity is the highest at acidic pH [10].

### 4. Effects of *N*-acylethanolamine hydrolysis inhibitors on N1E-115 cell viability

**Figure 3:**
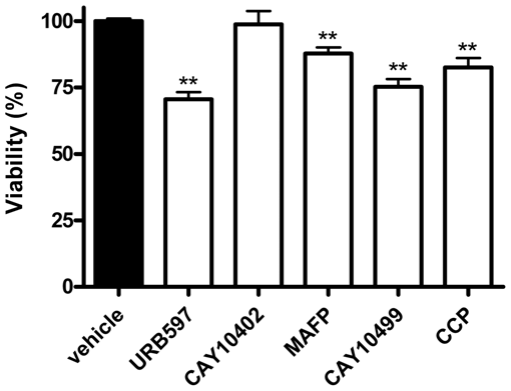
*N*-acylethanolamine hydrolysis inhibitors decrease N1E-115 cell viability. Cells were seeded 5h before treatment (2000 cells/well in microwells) and incubated with inhibitors at a concentration of 10 µM. After 72h of treatment, cytotoxicity was assessed by a MTT test. Data are the mean of three experiments performed in quintuplicate and are expressed as percentage of the vehicle control. Significantly different (**P<0.01) from vehicle incubation.

With these results in hand we moved on to evaluate the effects of the inhibitors alone, as well as these compounds in combination with the *N*-acylethanolamines, on cell viability. Thus we evaluated the cytotoxicity of these five inhibitors at 10 µM after 72 hours of incubation. While the reversible FAAH inhibitor CAY10402 did not provoke any cytotoxicity, the irreversible FAAH inhibitors URB597, MAFP and CAY10499 induced a significant decrease in cell viability (Fig. 3). Interestingly, these compounds were also the most potent at inhibiting AEA and PEA hydrolysis in intact N1E-115 cells (Table 2). The NAAA inhibitor CCP also significantly reduced cell viability, even though we were not able to detect its effects on *N*-acylethanolamine hydrolysis (Fig. 3).

**Figure 4:**
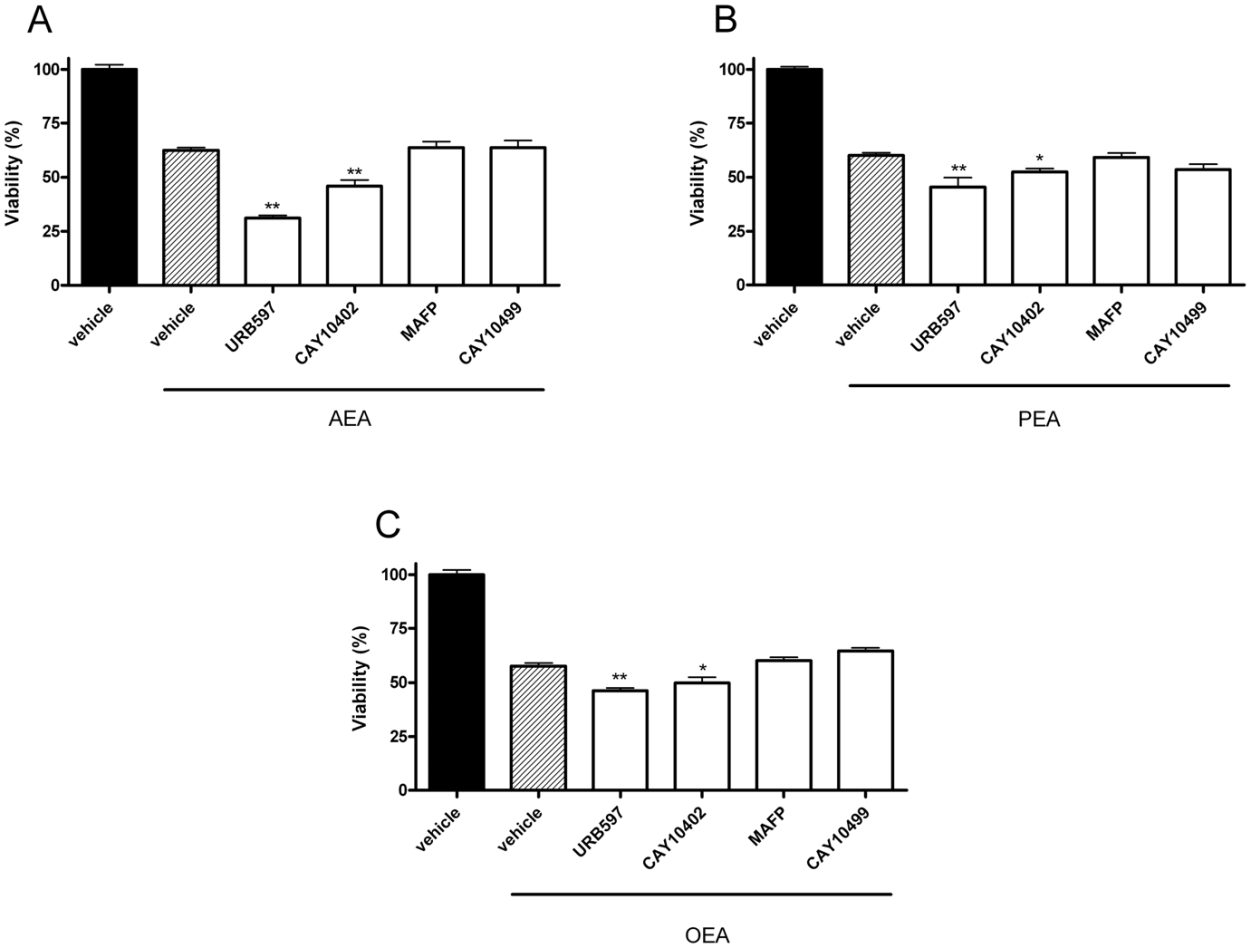
URB597 and CAY10402 potentiate *N*-acylethanolamine cytotoxicity. The FAAH inhibitors URB597 and CAY10402 potentiate AEA (*A*), PEA (*B*) and OEA (*C*) cytotoxicity. N1E-115 cells were seeded 5h before treatment (2000 cells/well in microwells) and incubated with the *N*-acylethanolamines (10 µM) with or without URB597, CAY10402, MAFP, or CAY10499 (at 10 µM). After 72h of treatment, cytotoxicity was assessed by a MTT test. Data are the mean of three experiments (performed in quintuplicate) and are expressed as percentage of the vehicle control. Statistical analysis were realized between each endocannabinoid alone compared to the endocannabinoid in presence of the inhibitor. Significantly different (*P<0.05; **P<0.01) from *N*-acylethanolamine incubation.

Next we co-incubated AEA, PEA and OEA (10 µM) with URB597, CAY10402, MAFP and CAY10499 (10 µM) to determine whether there would be an enhancement of the individual effects on cytotoxicity. Here we did not use CCP anymore because it was poor at inhibiting *N*-acylethanolamine hydrolysis in our cellular model. The reduction of cell viability produced by the *N*-acylethanolamines AEA, PEA and OEA was enhanced by the FAAH inhibitor URB597, with a much more pronounced response observed for the co-incubation of URB597 with AEA (Fig. 4A). A significant decrease in cell viability was also observed with the other selective FAAH inhibitor CAY10402 when incubated with AEA, PEA or OEA (Fig. 4A, 4B and 4C). Thus, for the next experiments we focused on the AEA-URB597 combination which, we found, produces the highest cytotoxicity (Fig. 4A).

### 5. AEA and URB597 co-incubation produces a decrease in N1E-115 cell proliferation without inducing cell apoptosis or necrosis

**Figure 5:**
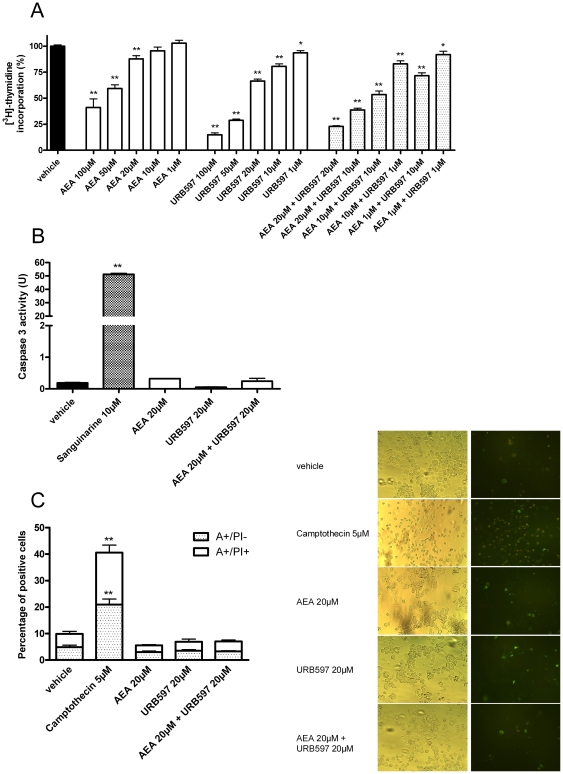
AEA and URB597 decrease cell proliferation without inducing apoptosis. N1E-115 cell proliferation was assed by [^3^H]-thymidine incorporation (*A*). The cells were seeded 5h before treatment (2000 cells/well in microwells) and incubated with increasing concentrations of AEA and/or URB597. After 16h of treatment, [methyl-^3^H]thymidine (0.5 mCi/well) was added during the last 8h of a 24h treatment. Data are the mean of three experiments (in quintuplicate) and are expressed as percentage of radiolabeled thymidine incorporation by vehicle control treated cells. Significantly different (*P<0.05; **P<0.01) from vehicle incubation. Caspase-3 activity (*B*) was measured in N1E-115 cells (4×10^5^ cells) after treatment with AEA, URB597 or a AEA-URB597 combination (20 µM, 24h). As a negative control, cells were treated with an equivalent volume of vehicle and a positive control was constituted by a 4h treatment with sanguinarine (10 µM). Data are expressed in pmoles of AFC (7-amino-4-trifluoromethylcoumarin) produced per min (U) (n = 3). Significantly different (**P<0.01) from vehicle incubation. Apoptosis was assessed by Annexin-V (A) staining and Propidium Iodide (PI) was used for the differentiation from necrosis (*C*). N1E-115 cells were treated with 20 µM of AEA and/or URB597 for 24h and the number of Annexin-V positive cells (A+/PI-) and of double stained cells (A+/PI+) was expressed as a percentage of total cells. Camptothecin (5 µM) was used as positive control. Data are the average of five random fields from experiments performed in triplicate. Significantly different (**P<0.01) from vehicle incubation.

With the aim to characterize the mechanism involved in the cytotoxicity of the AEA-URB597 combination, we first evaluated the influence of these molecules on N1E-115 cell proliferation and could observe a dose-dependent inhibition of [^3^H]-thymidine incorporation for the endocannabinoid and its metabolism inhibitor (EC_50_ = 45 µM and 31 µM for AEA and URB597 respectively) after 24h of treatment (Fig. 5A). Co-incubation of AEA at 10 µM and URB597 at 10 µM reduced cell proliferation by 50% compared to the vehicle control. Interestingly, URB597 alone decreased cell proliferation only by 21% at 10 µM, while AEA at the same concentration had practically no effect on cell growth. Similar results were obtained when AEA and URB597 were used at 20 µM and 1 µM alone or in a combination. Taken together these results suggest a synergistic action of the two compounds on N1E-115 cell proliferation.

By examining the cells after 24h, 48h or 72h of incubation with the tested compounds, almost no apoptotic cells were observed when looking at the morphology and when compared to the well-known apoptosis inducer sanguinarine (10 µM) (see Fig. S3).

No caspase 3 activity could be detected in our cells even after the 24 first hours of treatment with 20 µM of AEA and URB597 alone or in combination, whereas sanguinarine caused an increase in caspase 3 activity after 4 hours (Fig. 5B). Additionally, when looking at the percentage of apoptotic cells and necrotic cells, represented by annexin-V positive cells (A+/PI-) and double stained cells (A+/PI+) respectively, we could not observe any difference between cells treated with 20 µM of AEA and/or URB597 as compared to vehicle after 24h of incubation (Fig. 5C). We used camptothecin (5 µM, 6h incubation) as positive control for apoptosis induction and found a significant increase in the percentage of cells dying by both apoptosis (A+/PI-) and necrosis (A+/PI+). Note that similar results were obtained when incubating the cells for 72h instead of 24h (data not shown).

### 6. Alteration of N1E-115 cell cycle progression induced by AEA and URB597

**Figure 6:**
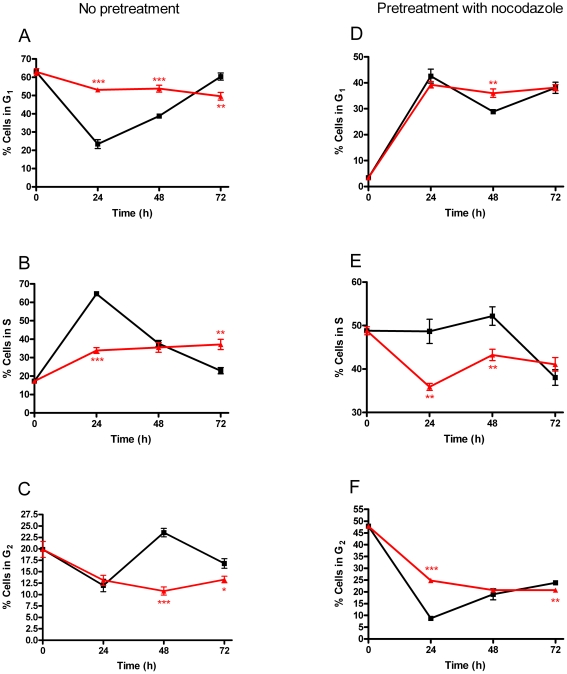
Alteration of N1E-115 cell cycle progression induced by AEA and URB597. Cells were seeded 5h before treatment (50, 100 or 150×10^3^ cells/well) and incubated for 24h, 48h or 72h respectively with vehicle (▪) or AEA and URB597 (▴) at 20 µM. Flow cytometric results show the percentages of cell population in G_1_ (*A*), S (*B*) and G_2_ (*C*). Part of cells were pretreated with nocodazole (30ng/ml, 14h) prior to the addition of AEA and URB597 and percentages of cell population in G_1_ (*D*), S (*E*) and G_2_ (*F*) was measured (n = 2). Significantly different (*P<0.05; **P<0.01; ***P<0.001) from vehicle incubation.

Since treatment of N1E-115 cells did not induce cell death, we hypothesized that the antiproliferative effect observed with AEA and URB597 might be the consequence of an arrest or slow-down in cell cycle progression. In agreement with the results obtained with [^3^H]-thymidine incorporation, we observed a significant accumulation of cells in G_1_-phase (53±0.5% for AEA-URB597; 23±2.4% for vehicle) (Fig. 6A), with a concomitant decrease in S-phase cells (34±0.6% for AEA-URB597; 65±1.1% for vehicle) (Fig. 6B), when comparing treated and untreated cells after 24h of incubation. However, when looking at cell cycle distribution after 48h and 72h of treatment, no additional accumulation of cells in G_1_-phase was noticed. The percentage of treated cells in G_2_-phase only decreased after 48h of treatment (Fig. 6C). Furthermore, we observed only small variations in cell cycle distribution of treated cells between the three times of measure, while untreated cells continued to progress through the cell cycle.

In addition, cells were pretreated with nocodazole in order to provoke mitotic arrest and accumulation of cells in G_2_-phase. In this case, when looking at cell cycle distribution following 24h of treatment with AEA-URB597 or vehicle, we observed no significant difference in the proportion of cells in G_1_-phase (Fig. 6D) and a smaller percentage in S-phase cells (Fig. 6E). A higher percentage of cells in G_2_-phase was noticed (Fig. 6F) though a overall decrease of their proportion in this phase throughout the treatment. We could then conclude that N1E-115 cells were not blocked in G_2_-phase.

Taken together, these results indicate a global slow-down of the cell cycle progression that appears to extend to all phases of the cell cycle. Nevertheless, we suggest a probable reduced transition through the G_1_/S checkpoint, leading to accumulation of cells in G_1_-phase, as displayed by the cell distribution observed after the first 24h of incubation.

### 7. Investigation of the potential molecular mechanism mediating *N*-acylethanolamine cytotoxicity on N1E-115 cells

**Figure 7:**
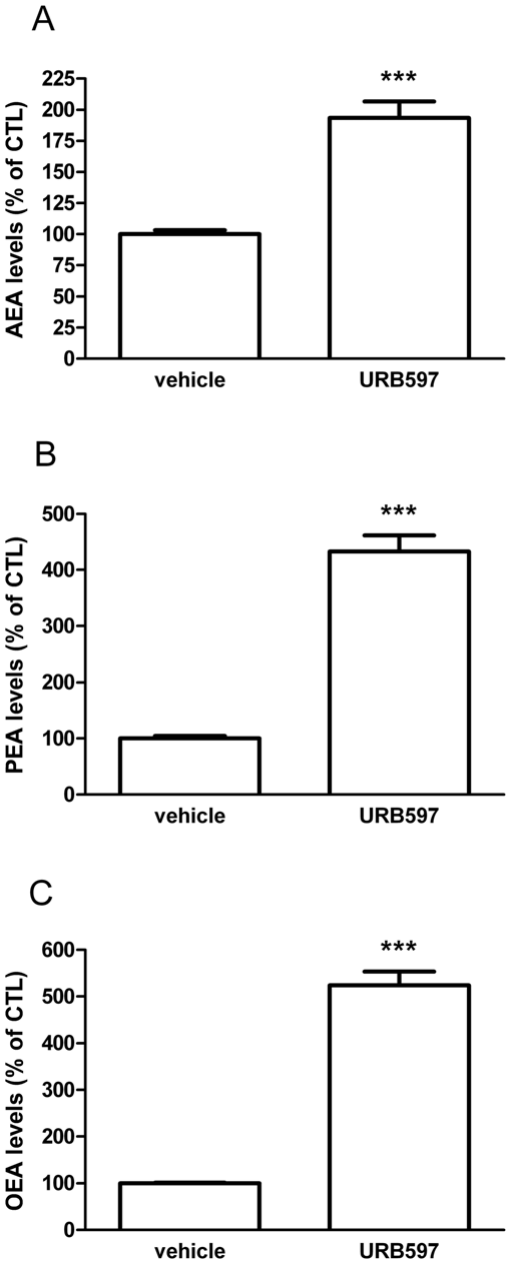
URB597 increases N1E-115 *N*-acylethanolamine levels. URB597 increases intracellular levels of AEA (*A*), PEA (*B*) and OEA (*C*) as measured by HPLC-MS. N1E-115 cells were seeded (12.5×10^6^ cells) and incubated for two hours with URB597 (1 µM). Data are the mean of three experiments performed in quadruplicate and are expressed as percentage of the vehicle control. Significantly different (***P<0.001) from vehicle incubation.

As URB597 and AEA both induce a decrease in cell proliferation, we first asked whether URB597 could act by increasing AEA levels. The aim of this experiment was to determine if URB597 could actually inhibit FAAH and modify AEA levels in our conditions. Thus we measured by an isotope-dilution HPLC-MS method the levels of AEA (as well as PEA and OEA) in N1E-115 cells. We found that incubating the cells with URB597 (1 µM, 2h) results in increased AEA levels up to 193% of the control (Fig. 7A). Note that, PEA and OEA levels were also enhanced (Fig. 7B and 7C).

**Figure 8:**
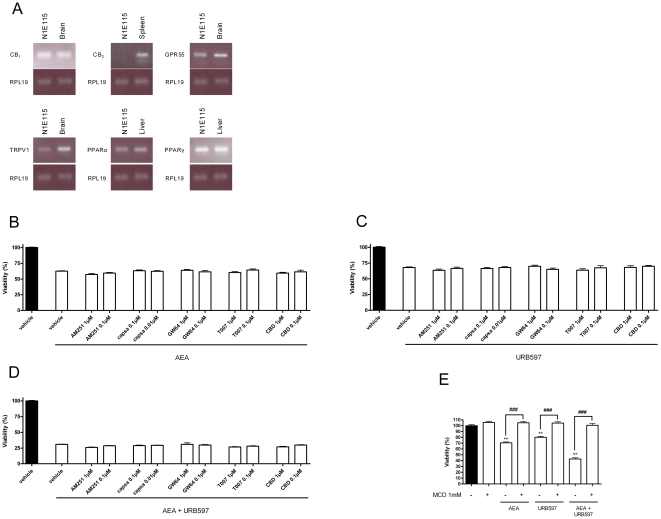
Investigation of the potential *N*-acylethanolamine molecular targets in N1E-115 cells. N1E-115 cells express cannabinoid receptor CB_1_ but not CB_2_, G-protein coupled receptor GPR55, vanilloid receptor TRPV1 and nuclear receptors PPARα and PPARγ (*A*). Detection of mRNA was performed by RT-PCR using mouse brain, spleen and liver as control. The blots are representative of three. Cytotoxicity of AEA (10 µM) (*B*), URB597 (10 µM) (*C*) and AEA + URB597 (*D*) was not significantly affected by CB_1_ receptor antagonist (AM251 - 0.1 and 1 µM), TRPV1 receptor antagonist (capsazepine - 10 and 100 nM), PPAR's receptor antagonists (GW6471 and T0070907 - 0.1 and 1 µM) and GPR55 receptor antagonist (cannabidiol, CBD - 0.1 and 1 µM). N1E-115 cells were seeded 5h before treatment (2000 cells/well in microwells) and incubated with AEA alone (10 µM), URB597 alone (10 µM) and combinations of these two molecules. Antagonists were added 1h prior to the addition of AEA and/or URB597. A MTT test was used to evaluate the percentage of viable cells remaining after 72h. Data are the mean of three experiments performed in triplicate and are expressed as percentage of the vehicle control. Disruption of lipid rafts inhibits AEA and URB597 mediated effects on N1E-115 cell viability (*E*). Cells were preincubated with methyl-β-cyclodextrin (MCD, 1mM) for 1h prior to the addition of 20 µM of AEA and/or URB597. Cell viability after 72h was assessed with a MTT test. Methyl-β-cyclodextrin had no effect by itself. Data are the mean of three experiments performed in triplicate and are expressed as percentage of the vehicle control. Significantly different (**P<0.01) from vehicle incubation. Significantly different (###P<0.001) from incubation without MCD.

To identify the molecular targets that could mediate the *N*-acylethanolamines / inhibitors cytotoxic effects, we looked by RT-PCR for the presence in N1E-115 of the reported *N*-acylethanolamine receptors. We confirm here that N1E-115 cells express the CB_1_, but not CB_2_ cannabinoid receptor (Fig. 8A) [25]. Other receptors implicated in *N*-acylethanolamine actions were also detected: the G protein-coupled receptor GPR55, the vanilloid cation channel TRPV1 and the nuclear receptors PPARα and PPARγ (Fig. 8A).

Based on the above data, we used selective antagonists in order to study the involvement of these receptors in AEA and/or URB597 cytotoxicity. Thus we used AM251, capsazepine, GW6471, T0070107 and cannabidiol, that are antagonists of the CB_1_, TRPV1, PPARα, PPARγ and GPR55 receptors, respectively. Cytotoxicity of AEA (10 µM), URB597 (10 µM) or both molecules co-incubation was not significantly affected by CB_1_ receptor antagonist (0.1 and 1 µM), TRPV1 receptor antagonist (10 and 100 nM), PPAR's receptor antagonists (0.1 and 1 µM) and GPR55 receptor antagonist (0.1 and 1 µM) (Fig. 8B, 8C and 8D). Similar observations were made when using smaller AEA and URB597 concentrations (data not shown). Note that the antagonist concentrations were chosen based on the literature and that the drugs were assayed alone to rule out the possibility that they could affect N1E-115 cell viability by themselves (see Fig. S4).

As we confirmed that AEA- and URB597-induced antiproliferative effects are not mediated by the classical molecular targets of this endocannabinoid, we next studied the possible implication of receptor-independent effects involving changes in cell membrane. We thus tested methyl-β-cyclodextrin (MCD, 1mM), a lipid raft disruptor frequently used to study the implication of these membrane microdomains in the cytotoxicity of numerous molecules such as cannabinoids. We saw here that MCD totally reversed the cytotoxicity mediated by AEA (10 µM), URB597 (10 µM) and both molecules co-incubation without modifying cell viability by itself (Fig. 8E).

## Discussion

The role of endocannabinoids in cancerogenesis has largely been explored in previous studies where they were mainly described as protective agents against tumor development. Indeed it has been shown using synthetic cannabinoid ligands that activating the cannabinoid receptors results in a reduction of cancer cell growth and tumor development. However, beside the strategy consisting of activating cannabinoid receptors using the exogenous administration of agonists, it is possible to activate those receptors by increasing the levels of endocannabinoids. Thus the aim of this study was to investigate the cytotoxicity of *N*-acylethanolamines in a neuroblastoma cell line and to potentiate their antiproliferative effects by inhibiting their hydrolysis.

As we expected, treatment of the neuroblastoma cell line N1E-115 with the endocannabinoid *N*-acylethanolamine AEA decreased cell viability. Indeed, the cytotoxicity of endocannabinoids on tumoral cells has been frequently reported. AEA has been shown to inhibit cholangiocarcinoma growth [30], to exert cytotoxic and antiproliferative effects on colorectal carcinoma cells [31], [32] and to cause apoptosis of osteocarcinoma cells [33] and glioma cells [16].

In our model, PEA and OEA also dose-dependently decreased cell viability. OEA cytotoxicity has been described as being the result of ceramide accumulation leading to cell apoptosis [34]. However, PEA was known to enhance the antiproliferative effect of endocannabinoids but not to exert cytotoxic effect by itself even at concentrations up to 10 µM [19], [35]. Here we clearly observed a dose-dependent cytotoxicity for PEA at concentrations below 10 µM. In order to evaluate if *N*-acylethanolamine degradation is indirectly responsible for cytotoxicity [31] we tested their acyl chain metabolites, arachidonic acid, palmitic acid and oleic acid and found that they were not responsible for the effect observed with the *N*-acylethanolamines.

As *N*-acylethanolamines are actually responsible for the cytotoxicity, we sought to investigate whether increasing their levels would affect the viability of our cells. Thus, we first looked for the presence of enzymatic activities degrading AEA and PEA in the neuroblastoma cell line. We detected an enzymatic hydrolysis for AEA, both in homogenates and in intact cells, that can be mostly attributed to the expression of FAAH. Similarly an enzymatic hydrolysis of PEA was also detected which can be explained, as for AEA hydrolysis, by the expression by the cells of FAAH but also NAAA mRNA. We then assayed several *N*-acylethanolamine hydrolysis inhibitors to see whether we could block their hydrolysis in cell homogenates and in intact cells. As expected all the inhibitors were less potent when tested on intact cells compared to cell homogenates. Nevertheless they were still able to significantly inhibit *N*-acylethanolamine degradation, with the exception of the NAAA inhibitor CCP. It is worth also mentioning that the reversible FAAH inhibitor CAY10402 was markedly less potent in inhibiting AEA hydrolysis compared to the irreversible inhibitors (URB597, CAY10499, and MAFP). This can be related to the larger effect of URB597 in reducing cell viability compared to CAY10402 (Table 2 and Fig. 3). One explanation for this lack of cytotoxicity might reside in the reversible character of FAAH inhibition by CAY10402, compared to the irreversible inhibition mediated by the other inhibitors tested, or an insufficient enzyme inhibition. The dual FAAH/MAGL inhibitors (MAFP and CAY10499) caused a significant decrease in cell viability that could be related to their ability to almost fully inhibit AEA hydrolysis at the tested concentrations. However, we have to keep in mind that these unselective inhibitors can also influence 2-AG hydrolysis via their action on the MAGL. Even though cytotoxic effects of 2-AG have also been found in many cell types, like prostate cancer [36], [37] or glioma [38], its antitumor properties are still controversial. Indeed, recent studies showed opposed events on cholangiocarcinoma growth where 2-AG acted like a growth-promoting agent, while AEA had antiproliferative effects [39], [40]. The conflicting actions of 2-AG have been evidenced when looking at the invasive properties of prostate carcinoma cells as well. Endogenous 2-AG was anti-invasive whereas cells treated with high doses of 2-AG saw their invasiveness enhanced [36]. However, we allow for the possibility that URB597 could also exert cytotoxic effects independent of its action on AEA hydrolysis.

To identify *N*-acylethanolamine - *N*-acylethanolamine hydrolysis inhibitor combinations that would increase cytotoxicity on N1E-115 cells, we tested AEA, PEA and OEA with inhibitors able to modify their hydrolysis in the same conditions. The incubation of cells with 10 µM of AEA and URB597 was found to be the most cytotoxic and reduced cell viability down to 30%. The FAAH inhibitor CAY10402 was slightly less efficient in improving AEA cytotoxicity maybe because of its reversible character compared to URB597. According to this, and to the fact that CAY10402 did not significantly decrease cell viability by itself, the decrease in cell viability caused by the combination AEA-URB597 might be in part attributed to the enhancement of AEA cytotoxicity. The same explanations could be held for the enhancement of PEA and OEA cytotoxicity by hydrolysis inhibitors. Indeed, they showed the same profile as AEA even though the effects were less marked, maybe because AEA is a better substrate for FAAH while NAAA is known to more selectively hydrolyze PEA [41]. Interestingly, the two dual inhibitors MAFP and CAY10499 did not affect *N*-acylethanolamine cytotoxicity even though they potently inhibited their hydrolysis in cultured cells and had cytotoxic effects by themselves. However, as described previously, these molecules are unselective and might interact with MAGL or other enzymes regulating lipid metabolism in the cell and therefore lead to less predictable effects.

We next sought to better characterize the mechanism of action of the combination AEA-URB597 as it was the most active in reducing N1E-115 cell viability. We showed that AEA and URB597 decreased [^3^H]-thymidine incorporation without inducing caspase-3 activation or increasing the percentage of apoptotic or necrotic cells. Treatment with AEA and URB597 slowed N1E-115 cell cycle progression and reduced transition through the G_1_/S checkpoint, causing accumulation of cells in G_1_-phase. Thus, we believe that the decrease in cell viability detected using the MTT assay consists in decreased cell proliferation rather than in cell death by apopotosis or necrosis. The antiproliferative properties of AEA have previously been shown at similar concentrations in rat glioma [17] but also in human colon cancer cells where AEA decreases polyamine levels [42], in human cholangiocarcinoma via activation of the non-canonical Wnt signaling pathway [30], and in human breast cancer and rat thyroid epithelial cancer cell lines via a modulation of expression and activity of key S phase regulatory proteins [13], [43], [44]. However, AEA has also been described as being an apoptosis inducer in many cell types like colorectal cancer, gastric cancer, osteosarcoma, glioma or prostate cancer cell lines [16], [33], [45]–[47]. Of note, depending on the method used, we observed slight differences in the effects of the tested molecules. Indeed, AEA was more effective in the MTT assay compared to the [^3^H]-thymidine assay. This could be attributed to the fact that cannabinoids, and more particularly AEA, were reported to affect mitochondrial function [48]–[51]. Since the MTT test consists in measuring mitochondrial succinate dehydrogenase activity of viable cells, the above described observations could explain the small variation obtained when comparing with [^3^H]-thymidine uptake at the same time and at the same concentration.

We show here that in our cell model, URB597 increases *N*-acylethanolamine intracellular concentrations, supporting the idea that its own cytotoxicity, and the ability of this FAAH inhibitor to enhance AEA antiproliferative effects, might be due to an influence on AEA levels but also of the other *N*-acylethanolamine levels. Indeed, these lipid mediators decrease cell viability by themselves but have also been described as being “entourage agents” potentiating AEA effects [35]. Furthermore, the implication of FAAH in cytotoxicity has been demonstrated in the liver where URB597 could enhance AEA-induced cell death [52]. Note that the discrepancy between the doses needed to obtain a reduction in cell proliferation when exogenously adding AEA (or OEA and PEA) and those obtained following FAAH inhibition by URB597 are not surprising. Indeed, it is well known that exogenously added endocannabinoids tend to stick to the culture plates thus reducing their bioavailability and therefore requiring higher concentrations than expected based on their affinity for their molecular targets. On the contrary, locally produced endocannabinoids (i.e. through inhibition of their catabolic enzymes) are readily available to interact with their target, thus explaining the lower concentrations needed to obtain a similar effect.

In order to elucidate the mechanism by which AEA and URB597 decrease cell viability, we used antagonists of the receptors for which mRNA was detected (CB_1_, TRPV1, GPR55, PPARα and PPARγ) to see whether we could block their antiproliferative effects. The lack of efficacy of these antagonists suggests that receptor-independent mechanisms are involved in the reduction of cell viability observed here. Indeed, we showed that the integrity of the lipid raft structure is required to mediate AEA and URB597 antiproliferative effects. Lipid rafts are specific membrane microdomains enriched in cholesterol playing a key role in membrane fluidity and protein trafficking [53]. They allow signaling molecules to concentrate and interact in order to facilitate signal transduction. Focusing on cancer biology, there is now growing evidence that lipid rafts are implicated in cell death, proliferation and migration [54], [55]. The role of lipid rafts in AEA cytotoxicity was previously described in HepG2 liver cancer cells and cholangiocarcinoma but, in both cases, these cytotoxic effects were also dependent of cannabinoid CB_1_ or CB_2_ receptors which could be controlled by these specific membrane microdomains [56], [57]. Similarly to what we observed here, DeMorrow et al. described lipid raft-mediated AEA cytotoxicity that was not prevented by cannabinoid receptor antagonists. Of note, AEA was described to induce cell death via an accumulation of ceramide and the recruitment of the Fas death receptor [39], although we observed here no induction of cell death but an inhibition of cell proliferation. Finally, we demonstrated here that URB597 exerts similar antiproliferative effects as AEA without inducing apoptosis or necrosis. Since we showed that this FAAH inhibitor increases the concentration of AEA and other *N*-acylethanolamines known to enhance AEA effects, we suggest that URB597 effects could be partly attributed to its ability to modulate AEA levels and then potentiate AEA effects on cell proliferation.

To conclude, we confirm here in a N1E-115 neuroblastoma model the antiproliferative effects of AEA. Additionally, we put into light the ability of URB597 to reduce cell proliferation, but not to induce apoptosis or necrosis, partly via enhancing *N*-acylethanolamine levels. This effect is independent of the known molecular *N*-acylethanolamine targets - cannabinoid, TRPV1, PPARα/γ or GPR55 receptors – activation, but relies on a lipid raft-mediated mechanism. Hence, the present report opens the way to potential *N*-acylethanolamine-based treatments aiming at reducing cancer cell proliferation through inhibition of their degradation.

## Supporting Information

Figure S1 **Arachidonic acid (AA), palmitic acid (PA) and oleic acid (OA) do not or only slightly decrease N1E-115 viability.** The cells were incubated with 0.1 µM, 1 µM, and 10 µM of AA, PA and OA. After 72h of treatment, cytotoxicity was assessed by a MTT test. Data are expressed as percentage of the vehicle control and are the mean of three experiments performed in quintuplicate. Significantly different (**P<0.01) from vehicle incubation.
(TIF)

Figure S2 **Structures of the endocannabinoid metabolism inhibitors used in this study**
(TIF)

Figure S3 **Morphology of N1E-115 cells after treatment with AEA and URB597.** N1E-115 cells do not die by apoptosis but still proliferate after treatment with AEA and URB597. Pictures of N1E-115 cells were taken after 24h, 48h and 72h of treatment with 20 µM of AEA, URB597 or a combination of both molecules, or with the vehicle control. Treatment of 4h with 10 µM of the inducing apoptosis compound sanguinarine was used to compare morphology.
(TIF)

Figure S4 **Cytotoxicity of receptor antagonists.** Cytotoxicity of CB_1_ receptor antagonist (AM251), TRPV1 receptor antagonist (capsazepine), PPARα and PPARγ receptor antagonists (GW6471 and T0070907 respectively) and GPR55 receptor antagonist (cannabidiol, CBD). N1E-115 cells were seeded 5h before treatment (2000 cells/well in microwells) and incubated with the antagonists. A MTT test was used to evaluate the percentage of viable cells remaining after 72h. Data are expressed as percentage of the vehicle control and are the mean of three experiments performed in quintuplicate.
(TIF)
