# Supplementary figures and images for: Increasing Antiproliferative Properties of Endocannabinoids in N1E-115 Neuroblastoma Cells through Inhibition of Their Metabolism
Source: PLoS One. 2011 Oct 27;6(10):e26823. doi: 10.1371/journal.pone.0026823 (PMC3203169; doi:10.1371/journal.pone.0026823)

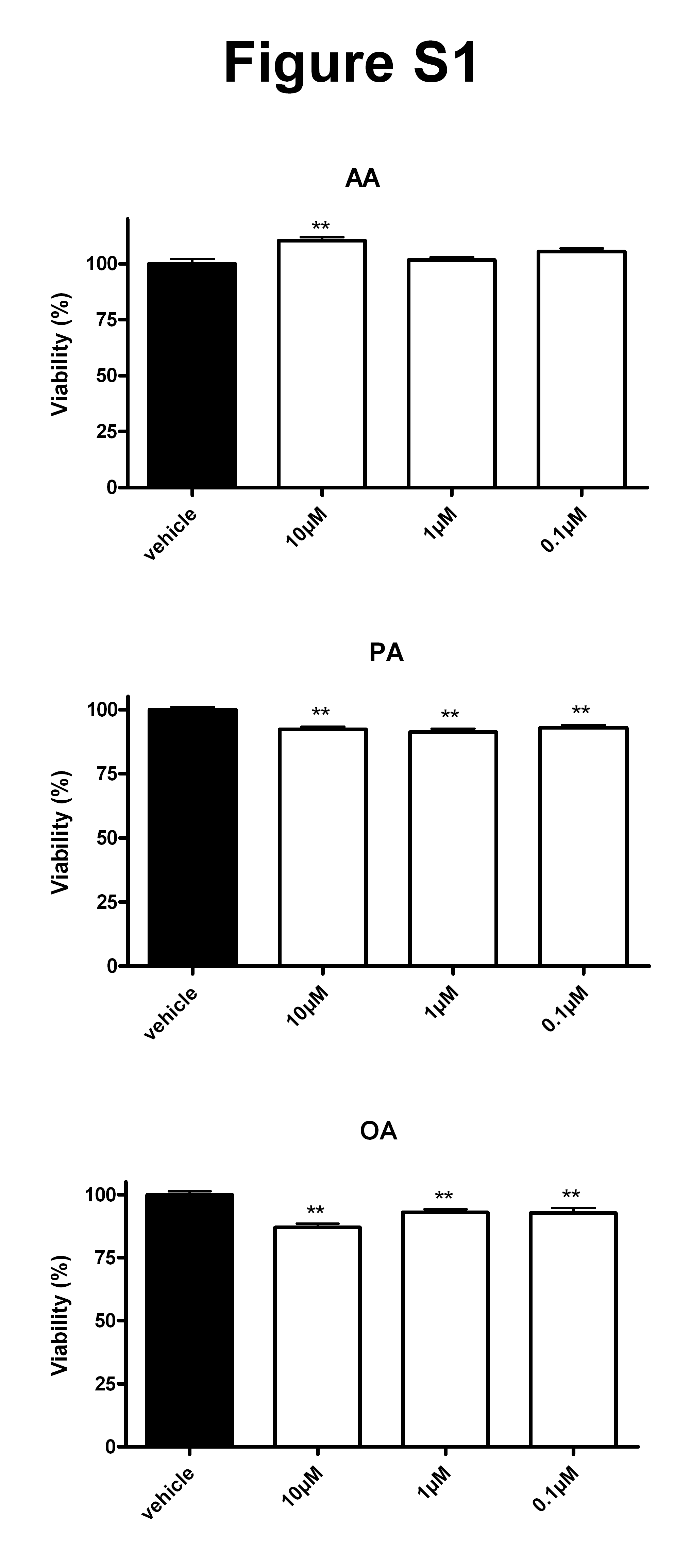

Supplement: Figure S1 — Arachidonic acid (AA), palmitic acid (PA) and oleic acid (OA) do not or only slightly decrease N1E-115 viability. The cells were incubated with 0.1 µM, 1 µM, and 10 µM of AA, PA and OA. After 72h of treatment, cytotoxicity was assessed by a MTT test. Data are expressed as percentage of the vehicle control and are the mean of three experiments performed in quintuplicate. Significantly different (**P<0.01) from vehicle incubation. (TIF) [file pone.0026823.s001.tif]

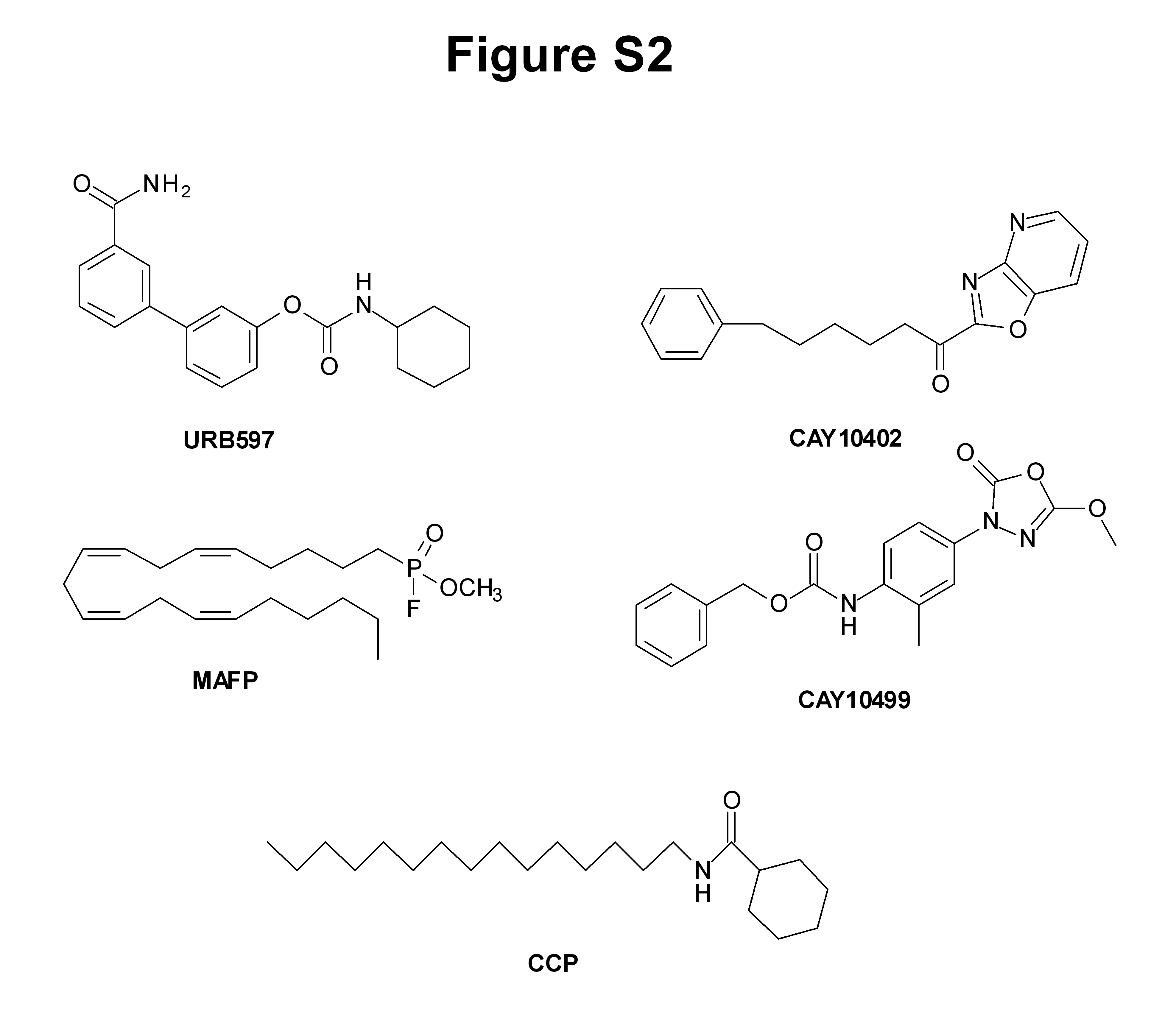

Supplement: Figure S2 — Structures of the endocannabinoid metabolism inhibitors used in this study (TIF) [file pone.0026823.s002.tif]

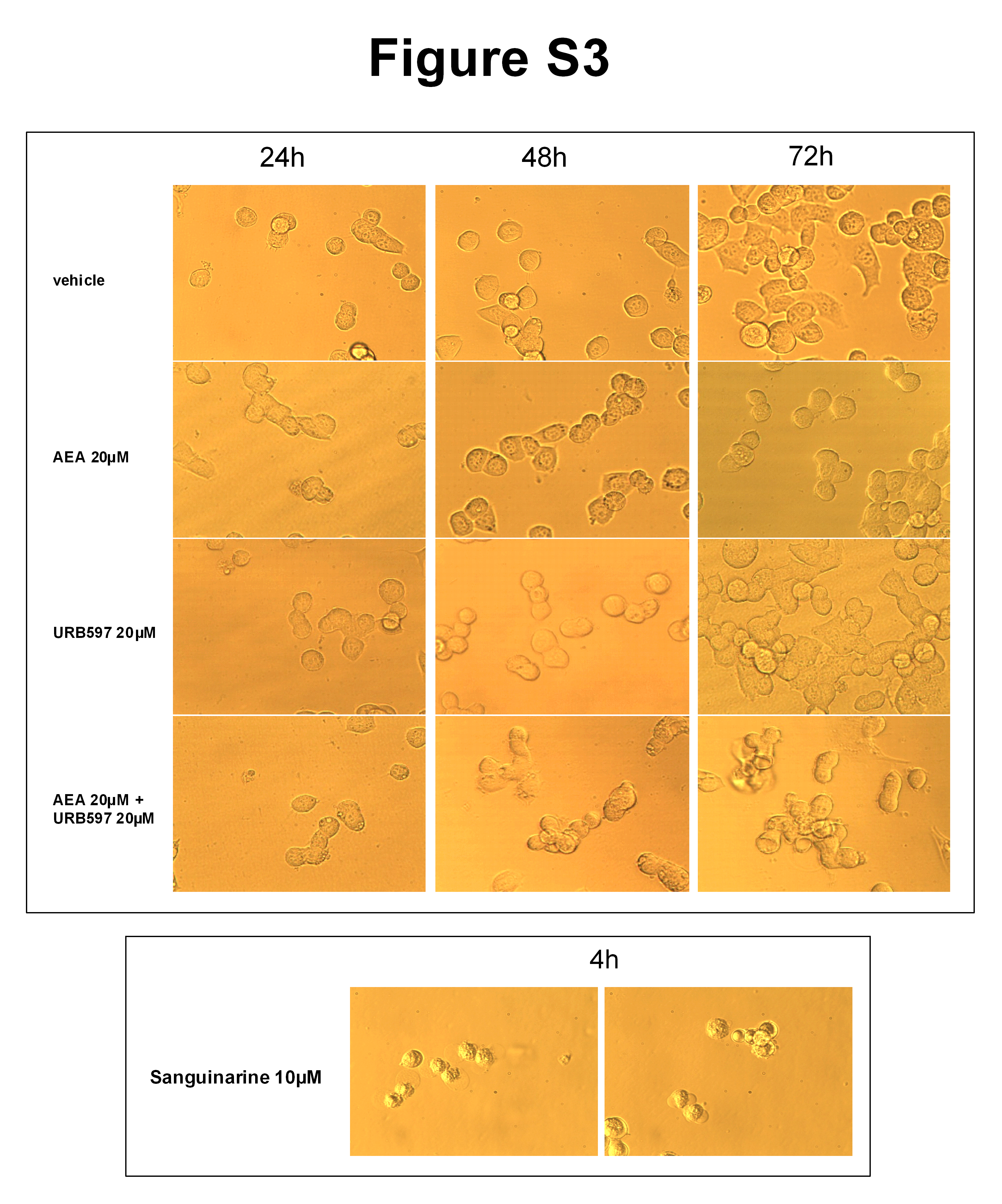

Supplement: Figure S3 — Morphology of N1E-115 cells after treatment with AEA and URB597. N1E-115 cells do not die by apoptosis but still proliferate after treatment with AEA and URB597. Pictures of N1E-115 cells were taken after 24h, 48h and 72h of treatment with 20 µM of AEA, URB597 or a combination of both molecules, or with the vehicle control. Treatment of 4h with 10 µM of the inducing apoptosis compound sanguinarine was used to compare morphology. (TIF) [file pone.0026823.s003.tif]

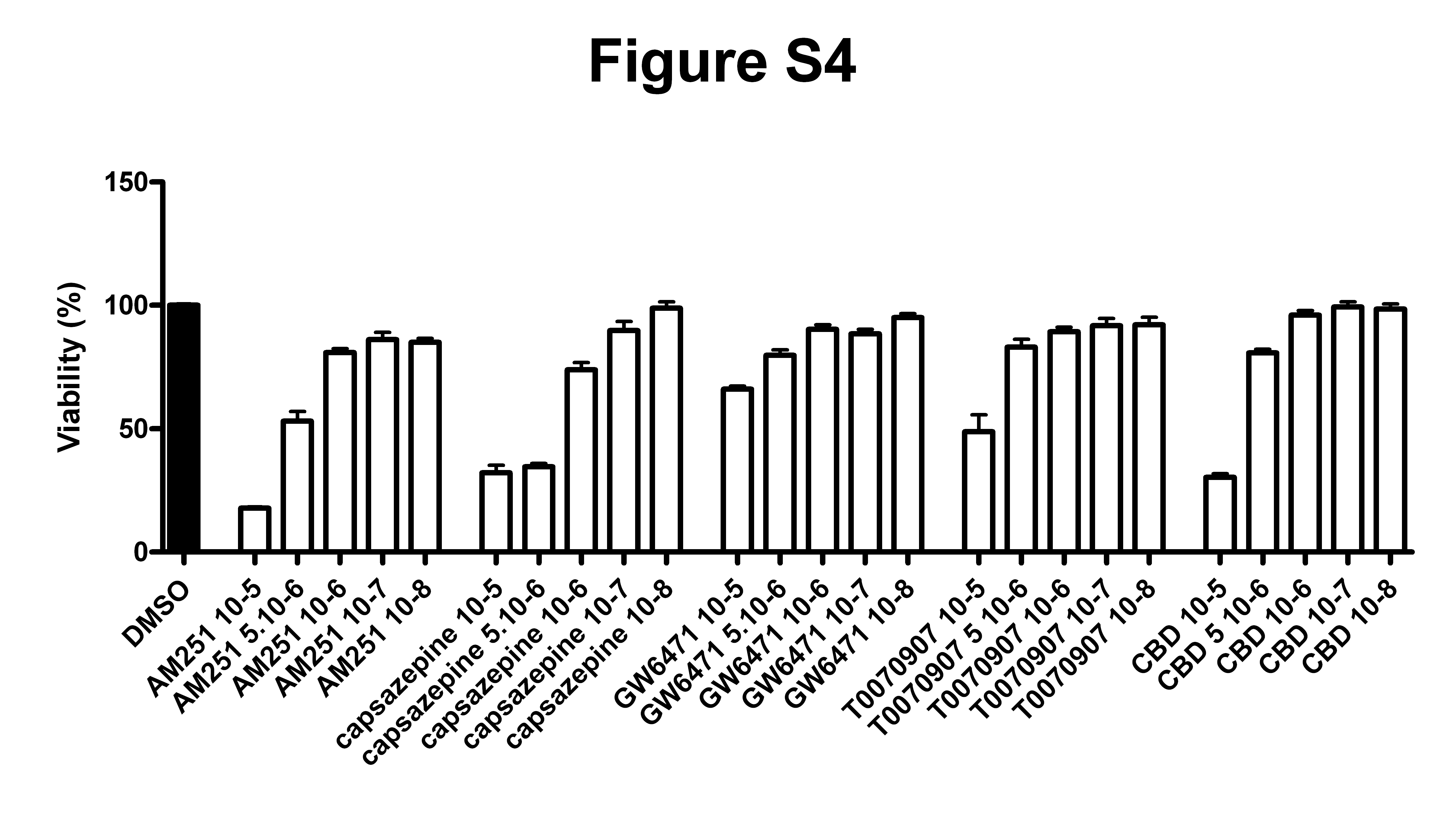

Supplement: Figure S4 — Cytotoxicity of receptor antagonists. Cytotoxicity of CB1 receptor antagonist (AM251), TRPV1 receptor antagonist (capsazepine), PPARα and PPARγ receptor antagonists (GW6471 and T0070907 respectively) and GPR55 receptor antagonist (cannabidiol, CBD). N1E-115 cells were seeded 5h before treatment (2000 cells/well in microwells) and incubated with the antagonists. A MTT test was used to evaluate the percentage of viable cells remaining after 72h. Data are expressed as percentage of the vehicle control and are the mean of three experiments performed in quintuplicate. (TIF) [file pone.0026823.s004.tif]
